# Characterization and identification of long-chain hydrocarbon-degrading bacterial communities in long-term chronically polluted soil in Ogoniland: an integrated approach using culture-dependent and independent methods

**DOI:** 10.1007/s11356-024-33326-6

**Published:** 2024-04-15

**Authors:** Amara Ukamaka Okoye, Ramganesh Selvarajan, Chioma Blaise Chikere, Gideon Chijioke Okpokwasili, Kevin Mearns

**Affiliations:** 1 Department of Microbiology, Faculty of Science, University of Port Harcourt, Port Harcourt, 500272 Nigeria; 2 Department of Environmental Science, Florida Campus, University of South Africa, Roodepoort, 1709 South Africa; 3 Laboratory of Extraterrestrial Ocean Systems (LEOS), Institute of Deep-Sea Science and Engineering, Chinese Academy of Sciences, Sanya, 572000 China

**Keywords:** Chronic oil pollution, Hydrocarbon degradation, Long-chain alkanes, Bioremediation, Microbial characterization, Ogoniland, Nigeria

## Abstract

Escalating oil consumption has resulted in an increase in accidental spills of petroleum hydrocarbons, causing severe environmental degradation, notably in vulnerable regions like the Niger Delta. Complex mixture of these hydrocarbons particularly long-chain alkanes presents unique challenges in restoration of polluted environment due to their chemical properties. This study aimed to investigate the long-chain hydrocarbon-degrading bacterial communities within long-term chronically polluted soil in Ogoniland, by utilizing both traditional cultivation methods and modern culture-independent techniques. Results revealed that surface-polluted soil (SPS) and subsurface soil (SPSS) exhibit significantly higher total organic carbon (TOC) ranging from 5.64 to 5.06% and total petroleum hydrocarbons (TPH) levels ranging from 36,775 ppm to 14,087 ppm, compared to unpolluted soil (UPS) with 1.97% TOC and 479 ppm TPH, respectively. Analysis of carbon chain lengths reveals the prevalence of longer-chain alkanes (C20-28) in the surface soil. Culture-dependent methods, utilizing crude oil enrichment (COE) and paraffin wax enrichment (PWE), yield 47 bacterial isolates subjected to a long-chain alkane degradation assay. Twelve bacterial strains demonstrate significant degradation abilities across all enriched media. Three bacterial members, namely *Pseudomonas* sp. (*almA*), *Marinomonas* sp. (*almA*), and *Alteromonas* (*ladA*), exhibit genes responsible for long-chain alkane degradation, demonstrating efficiency between 50 and 80%. Culture-independent analysis reveals that surface SPS samples exhibit greater species richness and diversity compared to subsurface SPSS samples. *Proteobacteria* dominates as the phylum in both soil sample types, ranging from 22.23 to 82.61%, with *Firmicutes* (0.2–2.22%), *Actinobacteria* (0.4–3.02%), and *Acidobacteria* (0.1–3.53%) also prevalent. Bacterial profiles at genus level revealed that distinct variations among bacterial populations between SPS and SPSS samples comprising number of hydrocarbon degraders and the functional predictions also highlight the presence of potential catabolic genes (*nahAa*, *adh2*, and *cpnA*) in the polluted soil. However, culture-dependent analysis only captured a few of the dominant members found in culture-independent analysis, implying that more specialized media or environments are needed to isolate more bacterial members. The findings from this study contribute valuable information to ecological and biotechnological aspects, aiding in the development of more effective bioremediation applications for restoring oil-contaminated environments.

**Supplementary Information:**

The online version contains supplementary material available at 10.1007/s11356-024-33326-6.

## Introduction

In an era marked by rapid technological advances and rising affluence, the global demand for fossil fuels, particularly oil and coal, has surged significantly. This escalating consumption has led to an increase in accidental spills of petroleum hydrocarbons, causing severe environmental degradation, notably in vulnerable regions like the Niger Delta (Nwilo and Badejo 2006; Ite et al. 2013; Okon and Ogba 2018). Crude oil is a complex mixture, predominantly composed of four categories: aliphatic (saturated) hydrocarbons, aromatics, asphaltenes, and resins (Cheng et al. 2014; Song et al. 2018). Within the aliphatics, alkanes exhibit a range of structural variants such as linear (n-alkanes), cyclic (cyclo-alkanes), and branched (iso-alkanes). Depending on the crude oil’s origin, alkanes can constitute a substantial 20–50% of its composition. Intriguingly, alkanes are also biosynthesized by diverse organisms like plants, algae, and bacteria for various biological functions such as waste management, structural support, and chemical signaling (Van Beilen et al. 2003). However, the degradation of these compounds, particularly long-chain alkanes, presents unique challenges due to their low solubility, minimal reactivity, and high solidification points, complicating both environmental remediation and efficient fossil fuel utilization (Rojo 2009; El Mahdi et al. 2016).

In the face of ongoing environmental pollution, the quest for sustainable and effective remediation techniques has never been more critical. Bioremediation emerges as an attractive and environmentally friendly alternative to traditional chemical and physical remediation methods, ensuring minimal disruption to the environment (Azubuike et al. 2016). This approach combines the natural capabilities of microorganisms to degrade pollutants into less harmful forms, prioritizing economic efficiency (Wu et al. 2023b). Among these microorganisms, bacteria play a pivotal role due to their metabolic versatility, capability for rapid reproduction, and resilience in extreme environmental conditions (Mrozik and Piotrowska-Seget 2010). Specifically, certain bacterial communities possess distinctive enzymatic systems capable of degrading hydrocarbons. For example, recent studies have explored the use of surfactants as a competitive alternative to enhance the removal of n-alkanes. While these systems have proven effective in the removal of various short-chain n-alkanes (Wu et al. 2022, 2023a) their efficacy for long-chain n-alkanes remains uncertain due to their low water solubility and bioavailability. This uncertainty highlights the ongoing challenge of detoxifying polluted soils and waters..The biodegradation of long-chain alkanes and paraffins has recently gained significant attention, particularly in diverse extreme environments, including long-term polluted soils. Therefore, understanding the composition and functional capabilities of these bacterial communities can open up avenues for efficient, cost-effective, and sustainable remediation strategies for long chain alkanes. Despite the urgency for effective bioremediation techniques, a significant gap exists in the comprehensive study of hydrocarbon-degrading bacterial communities, particularly in areas with a long history of chronic pollution. Existing research often leans either toward culture-dependent or culture-independent methodologies, failing to provide a holistic view of microbial diversity and functionality in contaminated environments (Das and Chandran 2011; Hedgpeth et al. 2021). Moreover, the mechanisms by which these microbial communities collectively degrade a broad spectrum of hydrocarbons, especially the more recalcitrant long-chain alkanes, are not fully understood (Head et al. 2006; Rojo 2009). Additionally, the genetic basis for hydrocarbon degradation, including the role of specific monooxygenase genes, is not fully elucidated. These gaps in knowledge signify a crucial need for an integrated approach that could offer more effective and adaptable solutions for soil remediation in chronically polluted areas.

Ogoniland, part of Nigeria’s Niger Delta, has been plagued by chronic pollution due to prolonged exposure to oil spills and leaks. This environmental catastrophe has not only jeopardized the local ecosystem but also the livelihoods of communities who depend on these lands for subsistence (Ogri 2001; Kponee et al. 2015). Given the complexity and resilience of hydrocarbon contaminants, especially long-chain alkanes, there is a pressing need to explore innovative and efficient cleanup strategies. One promising avenue is the study of naturally occurring hydrocarbon-degrading bacterial communities. These microbial assemblages in petroleum-contaminated environments have evolved mechanisms to utilize hydrocarbons as a carbon source (Austin and Callaghan 2013). This adaptation influences nutrient cycling, energy flow, and biogeochemical processes, presenting a potential bioremediation tool for contaminated sites (Chicca et al. 2022; Adetitun and Tomilayo 2023). The present work aimed to investigate the long-chain hydrocarbon-degrading bacterial communities within long-term chronically polluted soil in Ogoniland, by utilizing both traditional cultivation methods and modern culture-independent techniques. By combining enrichment strategies and genetic analysis of 16S rRNA gene sequences, the identification of hydrocarbonoclastic bacterial isolates was achieved. Additionally, selected bacterial strains were evaluated for their long-chain hydrocarbon degradation capabilities, alongside the characterization of flavin-binding monooxygenase genes (*alma* and *ladA*). In addition, we report bacterial strains proficient in efficiently breaking down a wide spectrum of hydrocarbons, including long-chain alkanes common in oil-polluted soils. Moreover, targeted amplicon analysis provided insights into the total bacterial populations and their functions in polluted and unpolluted soils, facilitating a comparative understanding of microbial communities in distinct environments.

## Materials and methods

### Sampling area

**Fig. 1:**
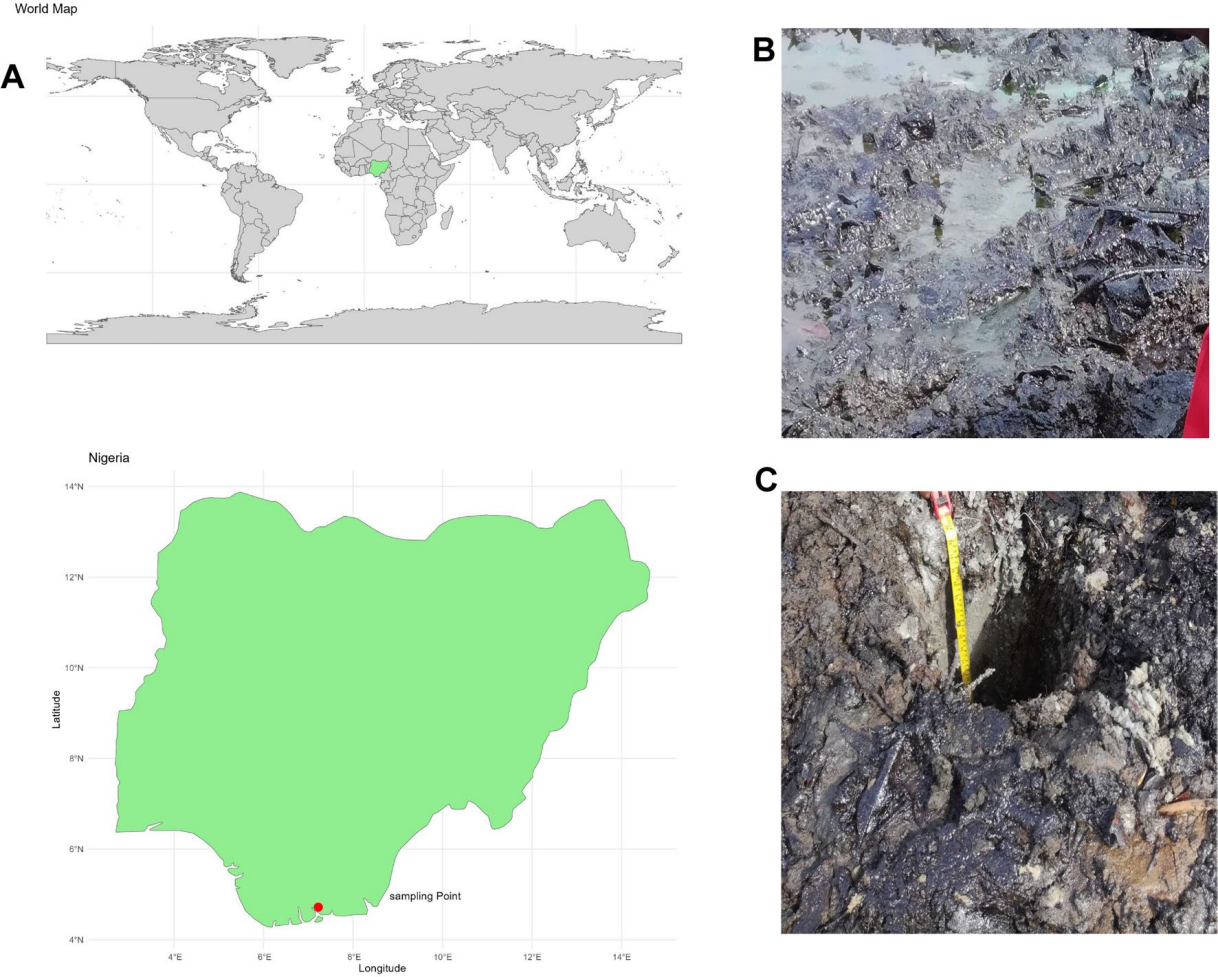
**A** Map highlighting the sample collection area in the Gio community of Niger Delta region, Nigeria, **B** surface soil impacted by oil contamination, and **C** sample collection from the subsurface soil layer (about 1 m depth)

Soil samples contaminated with crude oil were collected from four different points of North, South, West, and East—at a pollution site in Gio community, Tai Local Government Area, Niger Delta, Nigeria, as depicted in Fig. 1A. The selected sampling points is a zone of area heavily impacted by oil contamination, a consequence of accidental oil spill incidents. Sterilized soil augers were used for sample collection. Surface soil samples (SPS) were obtained from a depth ranging between 0 and 0.15 m, whereas subsurface soil samples (SPSS) were taken from a depth of 1 m, as illustrated in Fig. 1B–C. For control purposes, unpolluted soil samples (UPS) were collected approximately 1 km (km) away from the contaminated site (Supplementary Fig. 1). All samples were placed in sterile polyethylene bags and transported to the lab for analysis within 6 h, maintained at a temperature of 4 °C.

### Soil chemical properties

Soil chemical properties were analyzed for the collected soil samples. pH levels were monitored with a Metrohn automated probe analyzer (692, Herisau, Switzerland). Moisture content was measured by drying the soil at 105 °C for 12 h. The concentrations of nitrate (NO_3_^−^) and phosphate (PO_4_^−^), as well as toxic elements such as lead (Pb), nickel (Ni), and cadmium (Cd), were quantified following standard ASTM procedures and using ICP-OES, respectively. The electrical conductivity (EC) was determined using a conductivity meter, and the total organic carbon content was evaluated using the Walkley and Black (1934) method. The petroleum hydrocarbon content in the oil-contaminated soil was quantified using gas chromatography-flame ionization detector (GC-FID) analysis, adhering to the protocol given by Chikere et al. (2019). Briefly, for the extraction process, soil samples weighing 1 g each were combined with 20 ml of n-pentane solvent. This mixture allowed for ultra-sonication for 15 min, after which the samples were left to allow organic phase separation at 30 °C for 60 min. Subsequently, 1 ml of the separated organic phase was collected, transferred to GC vials, and prepared for analysis. The GC-FID apparatus was equipped with an HP 7673 FID detector, an autosampler, and a specialized fused silica capillary column. Both detector and injector were calibrated to temperatures of 320 °C and 250 °C, respectively. The oven’s temperature was set to start at 40 °C for 3 min, and then ramped up to 300 °C. Helium was used as the carrier gas, at a velocity of 38 cm/s. All data was processed using the Agilent Chemstation chromatography software (*v*10). The quantification of TPHs was attained by identifying the peak area, employing forced line integration via the Agilent Chemstation, spanning from n-C6 (n-hexane) to n-C35 (pentatriacontane).

### Enrichment, isolation, and biochemical characterization of hydrocarbon-degrading bacteria

To cultivate bacteria capable of degrading hydrocarbon, in particular long-chain alkanes in oil-contaminated soil, a 10 g soil sample was combined with 200 mL of modified Bushnell Hass (BH) Medium. This medium included the following components per liter: 0.2 g of magnesium sulfate (MgSO_4_), 0.02 g of calcium chloride (CaCl_2_), 1 g of monopotassium phosphate (KH_2_PO_4_), 1 g of dipotassium phosphate (K_2_HPO_4_), 1 g of ammonium nitrate (NH_4_NO_3_), and 0.05 g of ferric chloride (FeCl_3_) and 1 mL of trace element solution. The trace element solution, added at a rate of 1 mL per liter, consisted of 50 mg of MnCl_2_·H_2_O, 300 mg of H_3_BO_3_, 1.1 mg of FeSO_4_·7H_2_O, 190 mg of CoCl_2_·6H_2_O, 2 mg of CuCl_2_·2H_2_O, 24 mg of NiCl_2_·6H_2_O, 18 mg of NaMoO_4_·2H_2_O, 42 mg of ZnCl_2_·7H_2_O, and 1 mL of a vitamin solution (Pfennig and Trüper 1992). The pH of the medium was adjusted to 7.1, and it was sterilized at 121 °C for 20 min before adding the filter-sterilized vitamin solution. To further enrich the BH medium, 1 mL of Heavy Bonny crude oil (COE) and 1 g of paraffin wax (PWE) were added separately containing BH media to isolate bacteria capable of degrading long-chain alkanes. The mixture was then incubated at 30 °C and 100 rpm for 72 h. Cultures with an optical density at 660 nm > 1.0 were subsequently plated on agar containing BH medium and LB medium and incubated under the same conditions to obtain individual bacterial colonies of total hydrocarbon utilizing bacteria (THUB) and total heterotrophic bacteria (THB) respectively and the respective colony forming units (cfu) were calculated. Colonies displaying distinct morphological characteristics were selected and further purified through subculturing on Luria–Bertani (LB) plates. Isolates exhibiting distinct morphological traits, obtained from two separate enrichment media, were selected for a comprehensive array of biochemical analyses. These analyses encompassed assessments of catalase, oxidase, Methyl Red Voges Proskauer (MR-VP), citrate utilization, urease activity, and hydrogen sulfide (H_2_S) production. In addition, sugar fermentation tests, including glucose, lactose, and sucrose, were conducted to identify the bacterial members. The frequency of occurrence (%) was then calculated for both the COE and PWE sets of isolates. All isolated strains were preserved at -20 °C in liquid cultures containing 20% glycerol (*v/v*).

### Degradation assay for long-chain alkanes

To screen for hydrocarbon degradation capabilities, a little modified method adapted from Selvarajan et al. (2017a). The bacterial isolates, initially cultivated in enrichment media, were subsequently grown in BH media supplemented with glucose (10 g/L) and yeast extract (1.0 g/L). After a 72-h incubation period at 25 °C with continuous shaking at 120 rpm, cultures demonstrating robust growth were subjected to centrifugation at 10,000 × g for 10 min. The resulting pellet was resuspended in phosphate buffer, and this was followed by another centrifugation step to eliminate residual culture medium components. The final pellet was resuspended in phosphate buffer to an optical density matching the McFarland 0.5 standard. A 2,6-dichlorophenolindophenol (DCPIP) indicator assay was set up in a sterile 96-well microplate. Each well contained 20 µL of the bacterial suspension, 168 µL of BH medium, 12 µL of DCPIP, and 2 µL of a hydrocarbon source rich in long-chain alkanes (paraffin wax, hexadecane, and crude oil). In addition to the test wells, both glucose (10%) positive controls and blank negative controls were also added. These plates were incubated at 30 °C, and photometric readings at 600OD were taken after 72, 144, and 288 h of incubation.

### Molecular characterization and detection of potential gene in selected strains

Selected bacterial strains from both enrichment processes were subjected to molecular characterization for identification and testing their phytogenic relationship. DNA extraction from each culture was executed using the Quick g-DNA extraction kit (Zymo Research Corporation). This was followed by PCR amplification employing the 16S universal bacterial primers (27F and 1492R). The thermal cycling conditions included an initial denaturation at 95 °C for 5 min, then 32 cycles each of 95 °C denaturation for 30 s, 52 °C annealing for 30 s, and 72 °C elongation for 1 min, and with a final extension at 72 °C for 10 min. The PCR products were purified using gel purification kit and sent to Inqaba Biotech (Pretoria, South Africa) for sanger sequencing. The obtained sequences were then analyzed via BLAST to ascertain the identity of the isolates. Phylogenetic assessment employing the maximum-likelihood method was performed using the MEGA-X software (Centre for Evolutionary Medicine and Informatics, Tempe, AZ, USA) (Kumar et al. 2018). Functional gene identification (*almA* and *ladA* gene) was carried out by following the method given by Wang and Shao (Wang and Shao 2012) and Tourova et al. (Tourova et al. 2016) respectively.

### Total DNA extraction and Illumina sequencing

The genomic DNA from soil samples was extracted using the Power Soil™ DNA extraction kit as per the manufacturer’s instructions. To assess the quantity of extracted DNA, a Qubit fluorometer (Invitrogen, Carlsbad, CA, USA) was used. For DNA amplification, the PCR primer pairs 341F (CCTACGGGNGGCWGCAG) and 805R (GGACTACHVGGGTWTCTAAT) were employed, targeting the V3-V4 hypervariable region of the 16S rRNA gene. The DNA amplicons generated by PCR were confirmed using agarose gel electrophoresis. Initial purification of the amplicons was carried out using AMPure XP beads (Beckman Coulter, Brea, CA, USA) following the manufacturer’s instructions. After uniquely indexing the amplicons and adding Illumina sequencing adapters, an additional purification step was performed using AMPure XP beads. The resulting purified product was then normalized to ensure equal concentration, denatured, and loaded onto a MiSeq V3 cartridge for paired-end sequencing using the Illumina MiSeq sequencer (Illumina Inc., San Diego, CA, USA).

### Bioinformatics and diversity analysis

The raw Illumina MiSeq sequences underwent processing using QIIME2 (Bolyen et al. 2018) Platform. Before the QIIME2 analysis, the primers and adapter sequences were trimmed using cutadapt 3.1 (Martin 2011) and the quality of the sequences was assessed using FastQC (Andrews 2010). DADA2 (Callahan et al. 2016) was utilized for sequence denoising, eliminating low-quality reads, marginal sequences, and clustering sequences into amplicon sequence variants (ASVs). Then the ASVs were clustered with 100% similarity based on the representative sequences. The SILVA *v*138 database (Quast et al. 2012) served as the reference dataset for this process. Diversity analyses, including alpha and beta diversities, were calculated based on ASVs using QIIME2. To investigate the microbial community functional attributes in both polluted and unpolluted soil, Phylogenetic Investigation of Communities by Reconstruction of Unobserved States 2 (PICRUSt2) (Douglas et al. 2020) was employed. PICRUSt2 enables the prediction of functional profiles using 16S rRNA data from the community. The prediction outputs encompass enzymatic gene families and MetaCyc pathway profiles associated with the 16S rRNA representative sequences. Statistical analysis and visualizations were performed using Qiime2, Origin 2022, and R 4.0.2. All raw sequencing data have been deposited in the NCBI Sequence Read Archive (NCBI-SRA) under BioProject accession number PRJNA1037324.

## Results

### Chemical characteristics of polluted and unpolluted soil samples

The chemical characteristics were assessed across three distinct soil types: surface-polluted soil (SPS), subsurface-polluted soil (SPSS), and unpolluted soil (UPS), as detailed in Table 1. Among the pH levels, SPSS exhibited the acidic condition (pH 4.82), followed by SPS (pH 5.03), whereas UPS displayed relatively lesser acidity with a pH of 6.12. Temperature demonstrated slight variations between polluted and unpolluted soils. Regarding nutrient composition, nitrate concentrations were most prominent in SPS (8.33 mg/kg), followed by a decrease in SPSS (6.67 mg/kg), and the lowest levels were observed in UPS (3.84 mg/kg). Conversely, phosphate levels exhibited an inverse relationship, being highest in UPS (20.52 mg/kg) and lowest in SPS (8.11 mg/kg). Electrical conductivity (EC) reached its peak in UPS (185.46 µs/cm), succeeded by SPS (141.1 µs/cm) and SPSS (140.73 µs/cm). The trend of total organic carbon (TOC) inclined from SPS (5.64%) to SPSS (5.068%), reaching the lowest value in UPS (1.97%). Moisture content displayed its highest value in SPS (7.93%), followed by SPSS (6.76%), and the lowest content was recorded in UPS (4.88%). Concentrations of heavy metals like nickel, lead, and cadmium were generally more elevated in the polluted soils, though some exceptions were noted, such as nickel having the highest concentration in UPS (0.68 mg/kg).

**Table 1:** Chemical parameters of the collected soil samples

Parameters	Surface-polluted soil (SPS)	Subsurface-polluted soil (SPSS)	Unpolluted soil (UPS)
pH	5.03	4.82	6.12
Temperature ℃	29.3	27.6	30
Nitrate (NO_3_^−^)	8.33	6.67	3.84
Phosphate (PO_4_^−^)	8.11	10.834	20.52
EC (µs/cm)	141.1	140.73	185.46
Total organic carbon (TOC)%	5.64	5.068	1.97
Moisture content%	7.93	6.76	4.88
Nickel (Ni) mg/kg	0.38	0.36	0.68
Lead (Pb) mg/kg	0.54	0.41	0.0065
Cadmium (Cd) mg/kg	1.2	1.26	0.013

### Hydrocarbons

**Fig. 2:**
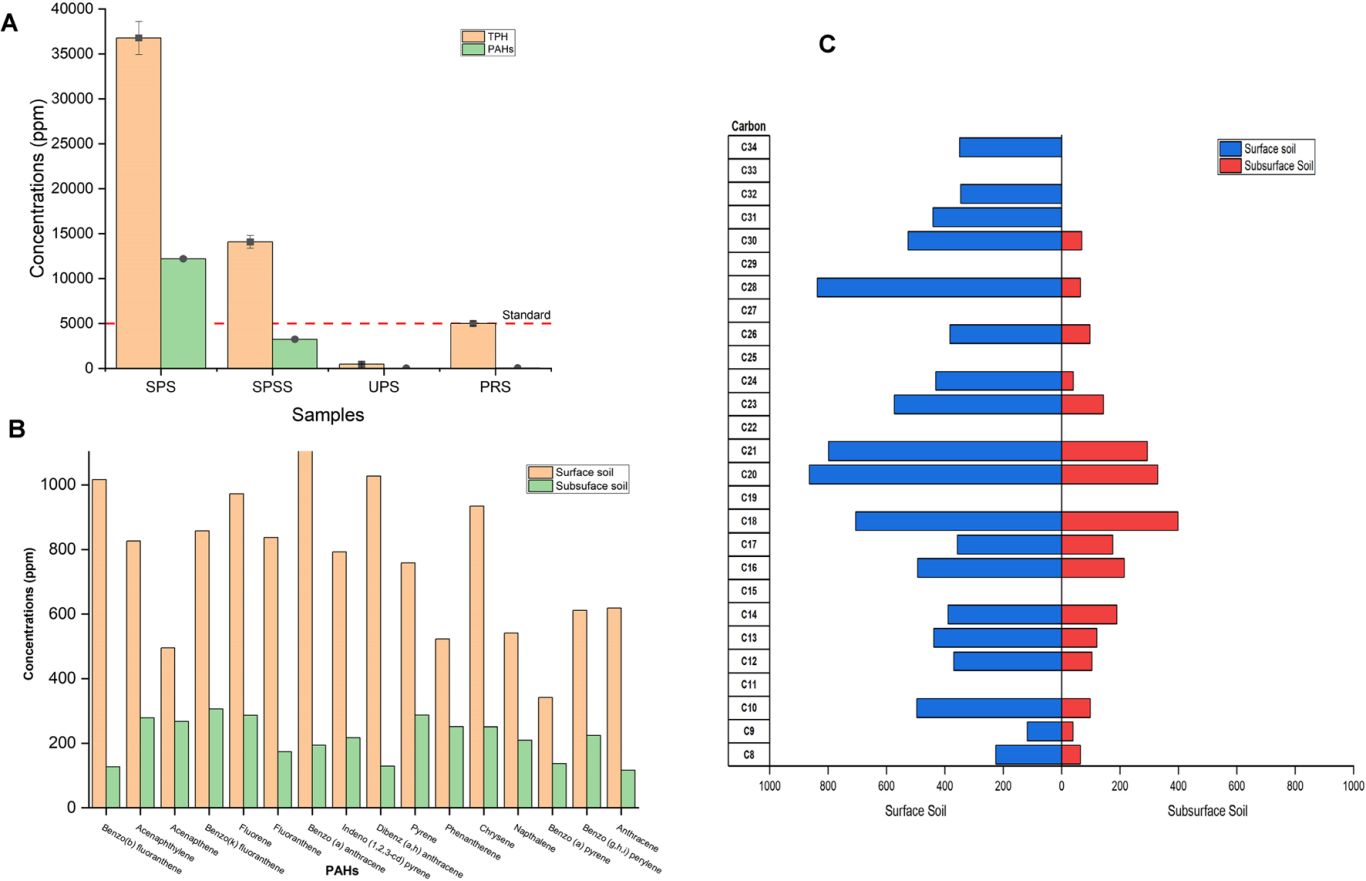
**A** Measured concentration (ppm) of total petroleum hydrocarbons (TPH) and polyaromatic hydrocarbons (PAHs) in oil polluted surface (SPS) and subsurface (SPSS) soil samples compared with unpolluted soil samples (UPS) and petroleum resource standard (PRS), **B** measured concentrations (ppm) of different PAHs, and **C** distribution of various carbon chain lengths in collected soil samples

The levels of total petroleum hydrocarbon (TPH) and polycyclic aromatic hydrocarbons (PAHs) in different soil samples were quantified (Fig. 2A); the results revealed that SPS showed the highest contamination, with TPH levels at 36,775 ppm and PAHs at 12,209.3 ppm. These were significantly higher than the levels in SPSS, which were 14,087.8 ppm for TPH and 3,248.75 ppm for PAHs. UPS exhibited minimal contamination, with TPH at 479.67 ppm and PAHs at 22.72 ppm, both far below the levels in polluted soils. For comparison, the petroleum resource standard (PRS) values were (5000 ppm for TPH and 40 ppm for PAHs) used and the findings indicate severe contamination in the surface- and subsurface-polluted soils, especially in the SPS, compared to both the unpolluted soil and established petroleum resource standards. An in-depth analysis of individual polycyclic aromatic hydrocarbons (PAHs) in both surface and subsurface contaminated soils were quantified, revealing a uniform pattern of elevated contamination levels in the surface soil. Significantly, benzo(a)anthracene was found to be the most abundant in the surface soil, with concentrations reaching 1120.2 ppm. In the subsurface soil, acenaphthylene was the most prevalent, recorded at 278.624 ppm. In contrast, the lowest levels were detected for benzo(a)pyrene in the surface soil, at 341.3 ppm, and for anthracene in the subsurface soil, measuring at 116.323 ppm (Fig. 2B).

The distribution of various carbon chain lengths (C8 to C34) in surface- and subsurface-polluted soils was investigated. Notably, the highest concentration in the surface-polluted soil was observed for C20, which recorded a level of 864.615 ppm, followed closely by C28 at 798.78 ppm. In contrast, the subsurface-polluted soil had its highest concentration at C18, registering at 398.126 ppm. Most notably, the surface soil consistently displayed higher concentrations of long-chain alkanes compared to the subsurface soil (Fig. 2C). Additionally, certain long-chain alkanes such as C19, C22, C25, C27, C29, C31, C32, and C33 were absent in both soil types, highlighting the specificity of alkane degradation or accumulation in these environments.

## Culture-dependent analysis

### Bacterial enrichment, isolation, and characterization

**Fig. 3:**
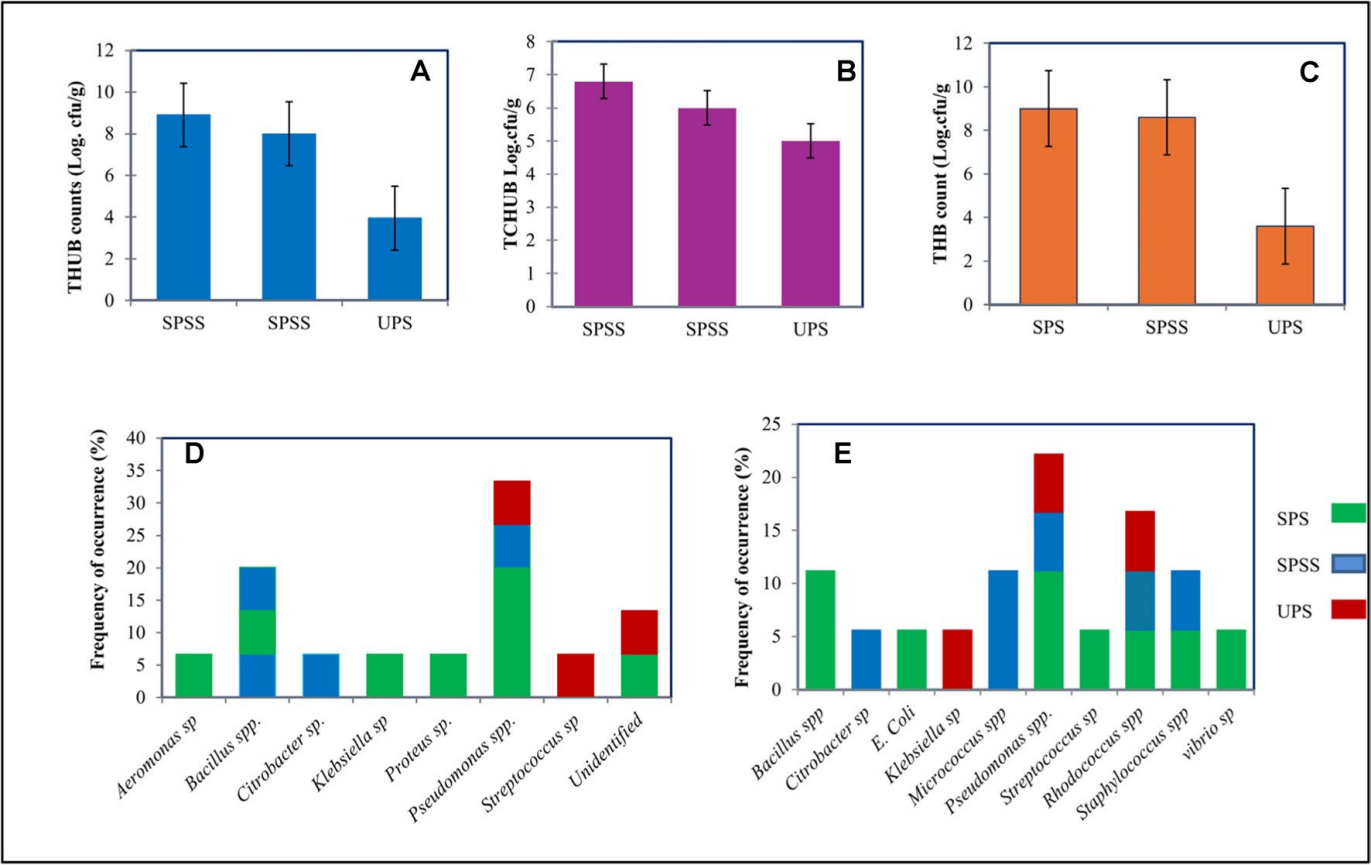
**A** Hydrocarbon utilization bacterial counts from crude oil enrichment (COE); **B** hydrocarbon utilization bacterial counts from paraffin wax enrichment; **C** the total heterotrophic bacterial counts (THB) of collected soils; **D** frequency of occurrence for bacterial isolates for paraffin wax enrichment; **E** frequency of occurrence for bacterial isolates for crude oil enrichment

The bacterial populations in collected soil samples were cultivated using two distinct enrichment methods: crude oil enrichment (COE) and paraffin wax enrichment (PWE). In SPS, the colony-forming units (cfu) of total hydrocarbon utilizing bacteria (THUB) were found to be 8.9 cfu/g and 6.8 cfu/g for COE and PWE, respectively. This was accompanied by a total of 9 cfu/g of total heterotrophic bacteria (THB). In SPSS, the counts were slightly lower, with THUB at 8 cfu/g (COE) and 6 cfu/g (PWE), alongside 8.6 cfu/g of THB. The UPS samples showed markedly lower bacterial counts, with THUB at 3.95 cfu/g (COE) and 5 cfu/g (PWE), and THB at 3.6 cfu/g (Fig. 3A–C).

Tables 2 and 3 present the biochemical characterization of bacterial isolates derived from enrichment media of crude oil and paraffin wax, respectively. A total of 47 pure bacterial colonies were isolated and subjected to a series of biochemical tests. The results shows that *Pseudomonas* sp. was the predominant bacterial type in the crude oil-enriched medium, followed by *Rhodococcus* sp., *Corynebacterium* sp., *Bacillus* sp., and *Micrococcus* sp. Conversely, *Proteus* sp., *Salmonella* sp., *E. coli, Serratia* sp., *Achromobacter* sp., *Providencia* sp., and *Vibrio* sp. were identified with lower frequency. Similarly, in the paraffin wax-enriched samples, *Pseudomonas* sp. emerged as the most frequently observed bacterial member, with subsequent occurrences of *Bacillus* sp., and *Achromobacter* sp. Other bacterial members, namely *Aeromonas* sp., *Rhodococcus* sp., *Serratia* sp., and *Acinetobacter* sp., were identified less frequently. In soil samples, variations in the frequency of bacterial members were evident depending on the specific enrichment conditions. In both types of enriched samples, the presence of bacterial members such as *Bacillus* sp., *Pseudomonas* sp., and *Citrobacter* sp. was consistently detected. Notably, paraffin wax-enriched samples displayed a distinctive set of bacterial members including *Aeromonas* sp., *Proteus* sp., and *Klebsiella* sp. (Fig. 3D), while crude oil-enriched media revealed a prevalence of different bacterial members, encompassing *Micrococcus* sp., *E. coli*, *Rhodococcus* sp., *Staphylococcus* sp., and *Vibrio* sp. (Fig. 3E). Additionally, surface samples consistently exhibited a higher frequency of bacterial members when compared to sub-surface and unpolluted soil samples. Among the identified bacterial members, *Pseudomonas* sp. demonstrated as the dominant species, present not only in enriched media but also across all collected samples.

**Table 2:** Biochemical characterization of bacterial members isolated from crude oil enrichment (COE)

Isolate code	Gram Rxn	Shape	oxidase	Citrate	MR	VP	Catalase	Indole	Urease	Motility	H_2_S/gas prod	Glucose ferm	Lactose ferm	Sucrose ferm	Tentative identity
CE 1	+	R	+	-	+	-	+	+	-	-	-/-	+	+	+	*Corynebacterium* sp.
CE 2	+	C	+	+	-	+	+	-	-	+	±	+	+	+	*Rhodococcus* sp.
CE 3	-	R	-	-	+	-	+	-	+	+	±	-	-	-	*Proteus* sp.
CE 4	-	R	+	+	-	-	+	-	+	+	-/ +	+	+	+	*Pseudomonas* sp.
CE 5	+	C	+	-	+	+	+	-	-	+	-/-	+	+	+	*Rhodococcus* sp.
CE 6	-	R	+	+	-	-	+	-	+	+	-/-	+	+	+	*Pseudomonas* sp.
CE 7	-	R	+	+	-	-	+	-	+	+	-/ +	+	+	+	*Pseudomonas* sp.
CE 8	+	R	-	+	+	-	+	+	-	-	-/ +	+	+	+	*Bacillus* sp.
CE 9	-	R	+	+	-	-	+	-	-	-	-/ +	+	-	-	*Salmonella* sp.
CE 10	-	R	-	-	-	-	+	+	-	-	-/ +	+	+	+	*E. coli*
CE 11	-	R	+	+	-	-	+	+	+	+	-/-	+	+	+	*Pseudomonas* sp.
CE 12	-	R	+	+	-	-	+	+	+	+	-/-	+	-	-	*Pseudomonas* sp.
CE 13	-	R	-	+	-	-	+	-	-	+	-/-	+	+	+	*Serratia* sp.
CE 14	-	R	+	-	-	+	+	-	-	-	-/ +	+	-	-	*Achromobacter* sp.
CE 15	+	C	+	+	+	+	+	+	-	-	-/-	+	-	-	*Micrococcus* sp.
CE 16	-	R	+	+	+	-	+	+	-	-	-/-	+	-	-	*Provideencia* sp.
CE 17	+	R	+	+	-	-	+	+	-	+	-/ +	+	-	-	*Bacillus* sp.
CE 18	-	R	+	+	+	-	+	-	-	-	-/ +	+	+	+	*Vibrio* sp.
CE 19	+	C	+	-	-	+	+	+	-	-	-/-	+	+	+	*Micrococcus* sp.
CE 20	+	R	+	-	-	-	+	+	-	-	-/ +	+	+	+	*Corynebacterium* sp.
CE 21	-	R	+	+	-	-	+	-	+	+	+ / +	+	-	-	*Pseudomonas* sp.

Key: *R*, rod; *C*, *cocci*; + , positive; -, *negative*; *MR*, methyl red; *VP*, Voges Proskauer; *ferm*., fermentation; *Rxn* = reaction; *prod*, production

**Table 3:** Biochemical characterization of bacterial members isolated from paraffin wax enrichment (PWE)

Isolate code	Gram Rxn	Shape	Oxidase	Citrate	MR	VP	Catalase	Indole	Urease	Motility	H2S/Gas prod	Glucose ferm	Lactose ferm	Sucrose ferm	Tentative identity
PWX1	+	R	-	+	+	-	+	-	-	+	-/-	-	-	-	*Aeromonas* sp.
PWX 2	-	R	+	+	-	-	+	-	-	+	-/-	-	-	-	*Pseudomonas* sp.
PWX 3	-	R	+	+	-	-	+	-	-	+	-/-	-	-	-	*Pseudomonas* sp.
PWX 4	-	R	+	+	-	-	+	-	-	+	-/-	-	-	-	*Pseudomonas* sp.
PWX 5	+	R	-	+	+	-	+	-	-	+	-/-	-	-	-	*Aeromonas sp*
PWX 6	-	R	+	+	-	-	+	-	-	+	-/-	-	-	-	*Pseudomonas* sp.
PWX 7	+	C	+	+	+	+	+	-	-	+	±	-	-	-	*Rhodococcus* sp.
PWX 8	+	R	+	+	-	-	+	-	-	+	-/-	-	-	-	*NI*
PWX 9	+	R	-	+	-	-	+	+	-	-	-/-	+	+	+	*Bacillus* sp.
PWX 10	-	R	+	+	-	-	+	-	-	+	-/-	-	-	-	*Serratia* sp.
PWX 11	-	R	+	-	+	-	+	-	-	+	-/-	-	-	-	*Acinetobacter* sp.
PWX 12	-	R	+	+	-	-	+	-	-	+	-/-	-	-	-	*Pseudomonas* sp.
PWX 13	-	R	+	+			+	-	-	+	±	+	-	-	*Pseudomonas* sp.
PWX 14	+	R	-	+	-	-	+	-	-	-	-/-	-	-	-	*Bacillus* sp.
PWX 15	+	R	-	+	-	-	+	-	-	-	-/-	+	+	+	*Bacillus* sp.
PWX 16	-	R	+	-	-	+	+	-	+	+	-/ +	+	-	-	*Achromobacter* sp.
PWX 17	-	R	+	-	+	-	+	+	-	-	-/-	+	+	+	*Pseudomonas* sp.
PWX 18	+	R	-	+	-	-	+	-	-	-	-/-	-	-	-	*Bacillus* sp.
PWX 19	+	R	-	+	-	-	+	-	-	-	-/-	+	+	+	*Bacillus* sp.
PWX 20	-	R	+	-	-	+	+	-	+	+	-/ +	+	-	-	*Achromobacter* sp.
PWX 21	-	R	+	-	+	-	+	+	-	-	-/-	+	+	+	*Pseudomonas* sp.
PWX 22	+	R	-	+	-	-	+	-	-	-	-/-	-	-	-	*Bacillus* sp.
PWX 23	+	R	-	+	-	-	+	-	-	-	-/-	+	+	+	*Bacillus* sp.
PWX 24	-	R	+	-	-	+	+	-	+	+	-/ +	+	-	-	*Achromobacter* sp.
PWX 25	-	R	+	-	+	-	+	+	-	-	-/-	+	+	+	*Pseudomonas* sp.
PWX 26	+	R	-	+	-	-	+	-	-	+	-/-	+	+	+	*Bacillus* sp.

Key: *R*, rod; *C*, cocci; + , positive; -, negative; *MR*, methyl red; *VP*, Voges Proskauer; *ferm*., fermentation; *Rxn*, reaction; prod, production; *NI*, not identified

**Fig. 4:**
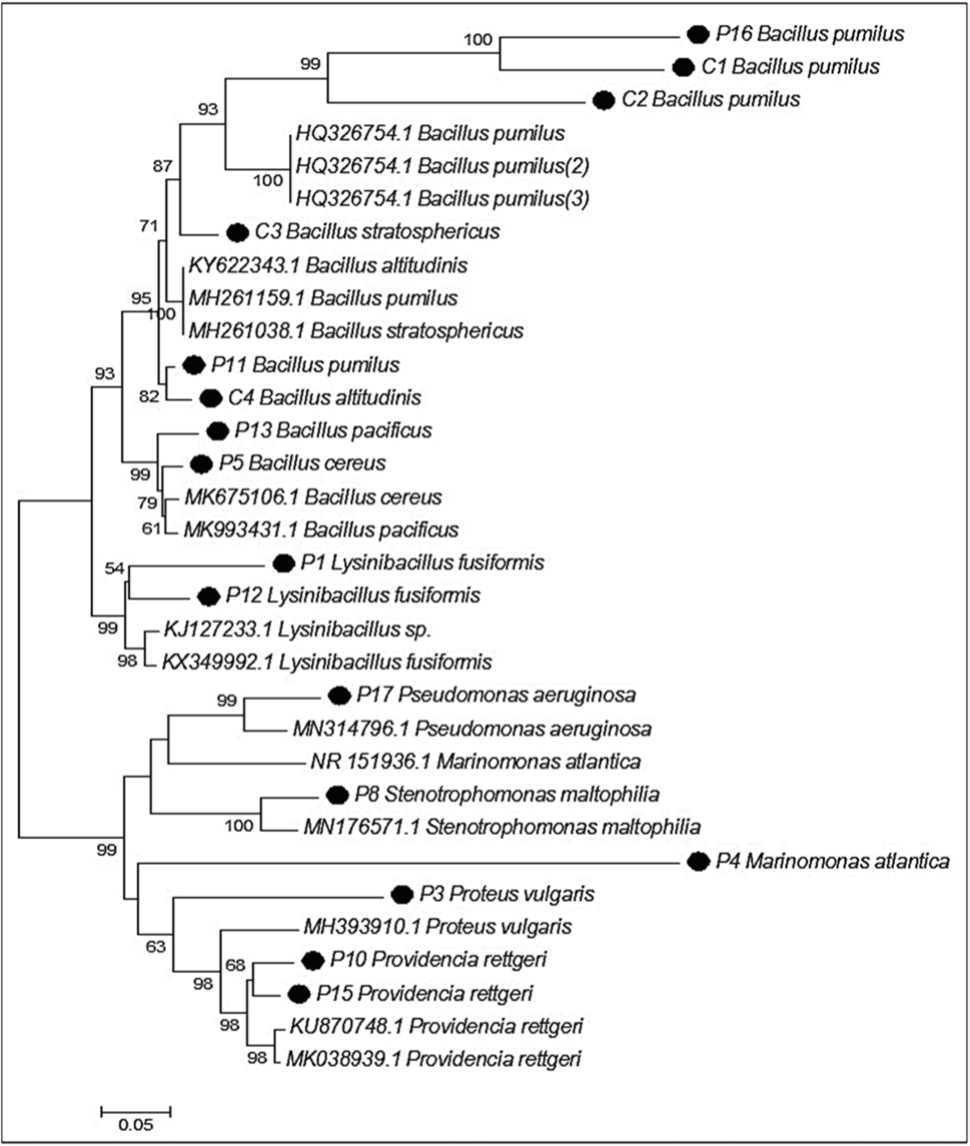
Phylogenetic tree was constructed using maximum likelihood based on 16S rDNA gene sequences, illustrating the bacterial isolates’ phylogenetic relationships. Each dot on the tree represents an isolated strain from this study. Strains labelled with codes “P” were isolated from paraffin wax enrichment, while those labelled “C” were isolated from crude oil enrichment

Following biochemical characterization, the bacterial strains were tested for initial growth on BH media enriched with glucose to optimize growth conditions. Out of the 47 isolates, 16 exhibited robust growth and were selected for subsequent molecular characterization and studies on the degradation of long-chain hydrocarbons. The analysis of PCR-amplified 16S rDNA sequences of these selected isolates, when compared with a reference species database from NCBI, revealed their affiliation with various bacterial genera. These genera included *Bacillus pumilis*, *Bacillus stratosphericus*, *Bacillus pacificus*, *Bacillus cereus*, *Pseudomonas aeruginosa*, *Streptromonas atlantica*, *Marinomonas atlantica*, *Proteus vulgaris*, and *Providencia rettgeri*. Importantly, all isolates exhibited a high nucleotide sequence similarity, ranging from 99 to 100%, with these known species. A phylogenetic tree, generated using MegaX software, is depicted in Fig. 4. This tree highlights that most of the isolates are classified into two phyla: *Firmicutes* and *Proteobacteria*. Within the *Firmicutes* cluster, isolates from the *Bacillaceae* family are notably prominent. Additionally, the phylogenetic tree highlights that all the isolates closely align with their respective genetic relatives.

### Degradation potential of long-chain alkanes

**Fig. 5:**
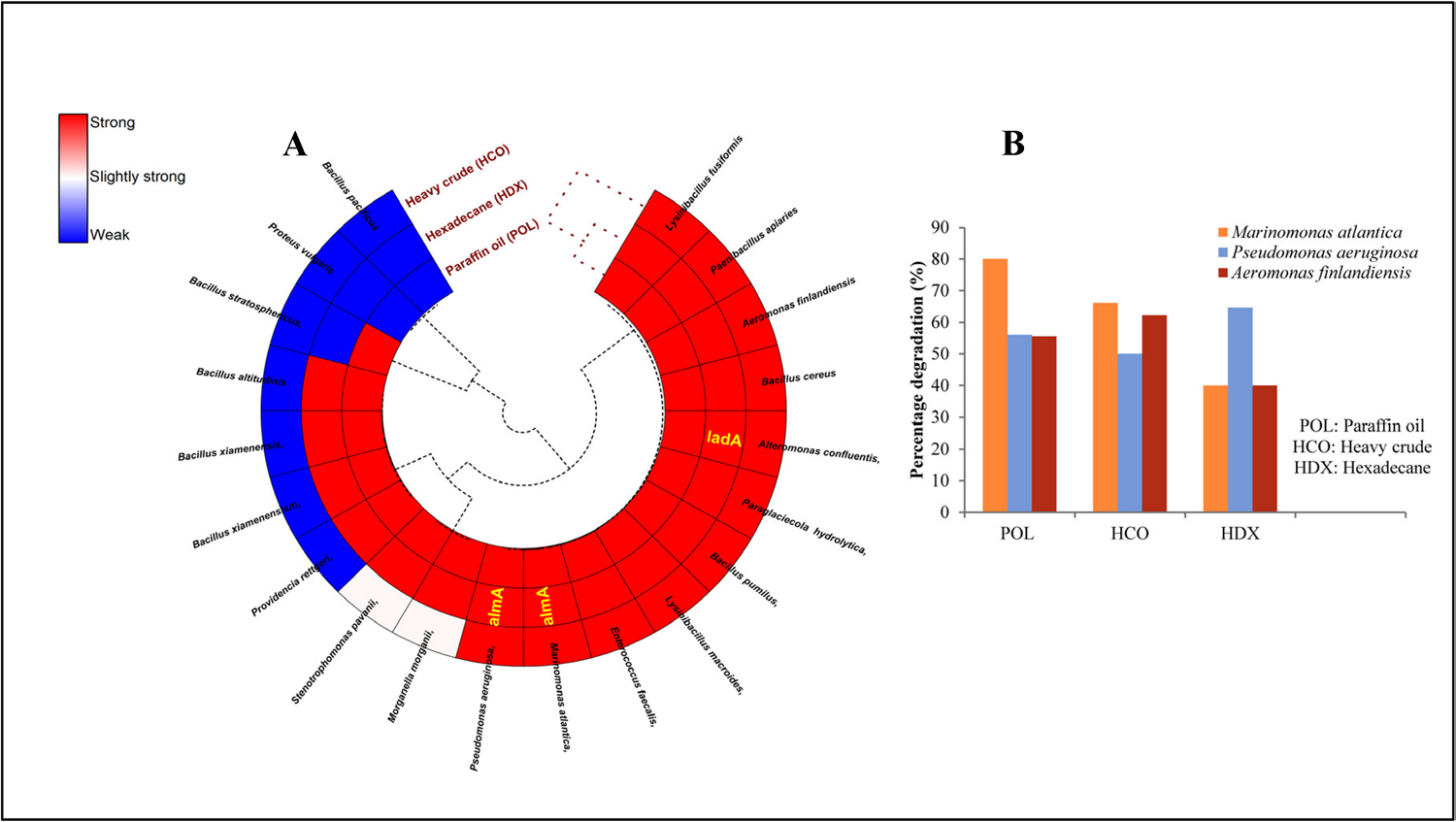
Long chain hydrocarbon degradation potential by selected bacterial members enriched with (**A**) paraffin oil, Hexadecane, and crude oil as sole carbon source—the presence of functional genes (*almA* and *ladA*) indicated in yellow with respect to the bacterial members. **B** Percentage of degradation by the bacterial members possessing functional genes

To evaluate the ability to degrade long-chain alkanes, about 20 bacterial strains were selected based on their growth conditions. These were then subjected to a degradation assay using BH medium with the DCPIP indicator, enriched with hydrocarbon sources rich in long-chain alkanes: paraffin wax (POL), hexadecane (HDX), and heavy crude oil (HCO). The experiment also included positive controls and incubated for a period of 288 h. The results revealed that 12 of the bacterial strains exhibited significant degradation abilities across all enriched media (Supplementary Table 1, Fig. 5A). However, a few bacterial members demonstrated weak degradation abilities in HCO-enriched media. Notably, strains like *Proteus* and *Bacillus pacificus* displayed no degradation capabilities against long-chain alkanes. To identify the functional genes responsible for degrading long-chain alkanes, the strains were subjected to targeted PCR analysis, specifically looking for the *almA* and *ladA* genes. Out of all the strains, only three bacterial members exhibited these target genes: *almA* was found in *Pseudomonas aeruginosa* and *Marinomonas atlantica*, whereas *ladA* was identified in *Alteromonas confluentis*. A subsequent analysis of these three strains’ degradation abilities revealed *Marinomonas* (POL (80%), HCO (66.2%) and HDX: (40%) as the most capable at degrading paraffin wax and crude oil. In contrast, *Pseudomonas* exhibited strong degradation efficiency (POL: 56%, HCO: 50%, HDX: 64.7%) against Hexadecane (Fig. 5B).

## Culture-independent analysis

**Fig. 6:**
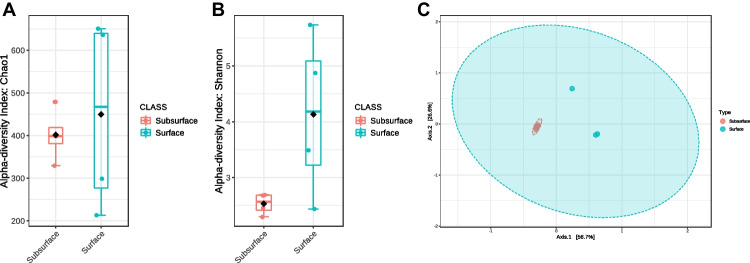
Alpha and beta diversity indices **A** Chao1 and **B** Shannon index, and **C** principal coordinate analysis (PCoA) plot based on Bray–Curtis dissimilarities of collected surface and subsurface soil samples

In total 8 soil samples collected from surface and subsurface of the oil polluted sites were sequenced using illumina platform. To evaluate community richness and diversity, α-diversity indices at the amplicon sequence variant (ASV) level were analyzed using Qiime 2. Examining the Chao1 diversity indices, surface samples exhibited greater species richness compared to subsurface samples (Fig. 6a). However, it is worth noting that the observed differences in bacterial richness did not reach statistical significance (*p*-value: 0.70559). Similarly, the Shannon index (Fig. 6b) indicated higher species diversity in surface soil samples compared to subsurface soil (*p*-value: 0.11533). Analyzing the Bray–Curtis dissimilarity between soil bacterial communities revealed differences in the community structure between surface and subsurface soil (Fig. 6c). Notably, subsurface samples tended to cluster together, while surface samples displayed some variations and clustered separately. Additionally, the observed differences in bacterial community composition between surface and subsurface samples in multivariate space were not highly significant (PERMANOVA *F-*value: 3.051; *R-*squared: 0.33709; *p*-value: 0.082).

**Fig. 7:**
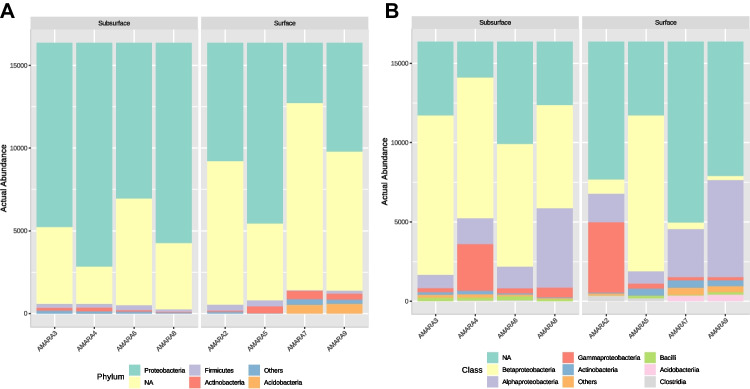
Abundance of phylotypes at the taxonomic rank **A** Phylum and **B** Class level observed in surface and subsurface soil samples

*Proteobacteria* emerged as the dominant phylum in both soil sample types, ranging from 22.23 to 66.71% in surface samples and from 57.49 to 82.61% in subsurface samples. In the surface soil samples, following *Proteobacteria*, *Acidobacteria* was prevalent, ranging from 0.1 to 3.53%. *Actinobacteria* (0.4 to 3.02%), *Firmicutes* (0.29 to 2.22%), and AD3 (0.1 to 1.56%) were also notably abundant (Fig. 7a). Conversely, in the subsurface soil samples, the abundance of *Firmicutes* (0.9 to 1.88%), *Actinobacteria* (0.36 to 1.38%), and *Acidobacteria* (0.1%) was comparatively lower than in the surface soil samples. Notably, both soil samples showed a high number of unclassified reads, indicating the potential presence of novel microbes in these polluted areas. Within the *Proteobacteria* phylum, the class *Betaproteobacteria* dominated in surface soil samples, ranging from 1.5 to 60.04%. It was followed by *Alphaproteobacteria*, which accounted for 4.76 to 37.46%, and *Gammaproteobacteria*, with an abundance ranging from 1.2 to 27.09%. In contrast, subsurface soil samples showed a higher abundance of *Betaproteobacteria*, ranging from 39.63 to 61.43%, compared to surface soil. The higher abundance of *Betaproteobacteria* in subsurface soil could be influenced by specific adaptations to subsurface conditions or the availability of substrates favoring the proliferation of this bacterial class. *Alphaproteobacteria* accounted for 5.06 to 30.65%, and *Gammaproteobacteria* were present at lower abundance, ranging from 1.58 to 17.96%, compared to surface soil samples. Additionally, both soil samples contained notable classes such as *Acidobacteriia* and *Bacilli* in a considerable abundance compared to other bacterial classes (Fig. 7b) suggests their potential ecological significance in hydrocarbon-contaminated environments, possibly playing roles in bioremediation processes or responding to the presence of long-chain alkanes.

**Fig. 8:**
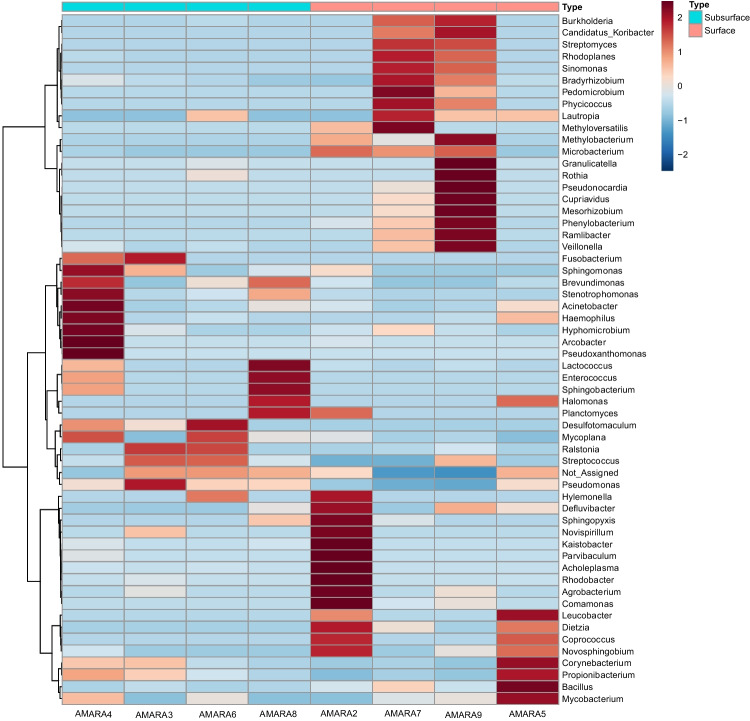
Heat map showing the dominant bacterial members (genus level) in the surface and subsurface soil samples

Clear distinctions between surface and subsurface soil samples were evident at the genus level. Surface samples exhibited a notable presence of genera including *Burkholderia, Pedomicrobium*, *Methyloversatilis*, *Rothia*, *Granulicatella*, *Deflivibacter*, *Rhodococcus*, *Rhodobacter*, *Agrobacterium*, *Novosphingbium*, *Bacillus*, and *Mycobacterium*. In contrast, subsurface samples were characterized by the prevalence of genera such as *Sphingomonas*, *Acinetobacter*, *Arcobacter*, *Hyphomicrobium*, *Halomonas*, *Mycoplana*, *Ralstonia*, *Pseudomonas*, and *Lactococcus*. It is worth noting that several genera were common to both sample types imply their versatility and adaptability to different soil layers, showcasing their potential role as key players in hydrocarbon degradation across the entire soil profile. The heatmap representation visually highlights the distribution patterns of these major genera (Fig. 8), emphasizing their relative abundance and providing a comprehensive overview of the microbial landscape in response to chronic pollution.

**Fig. 9:**
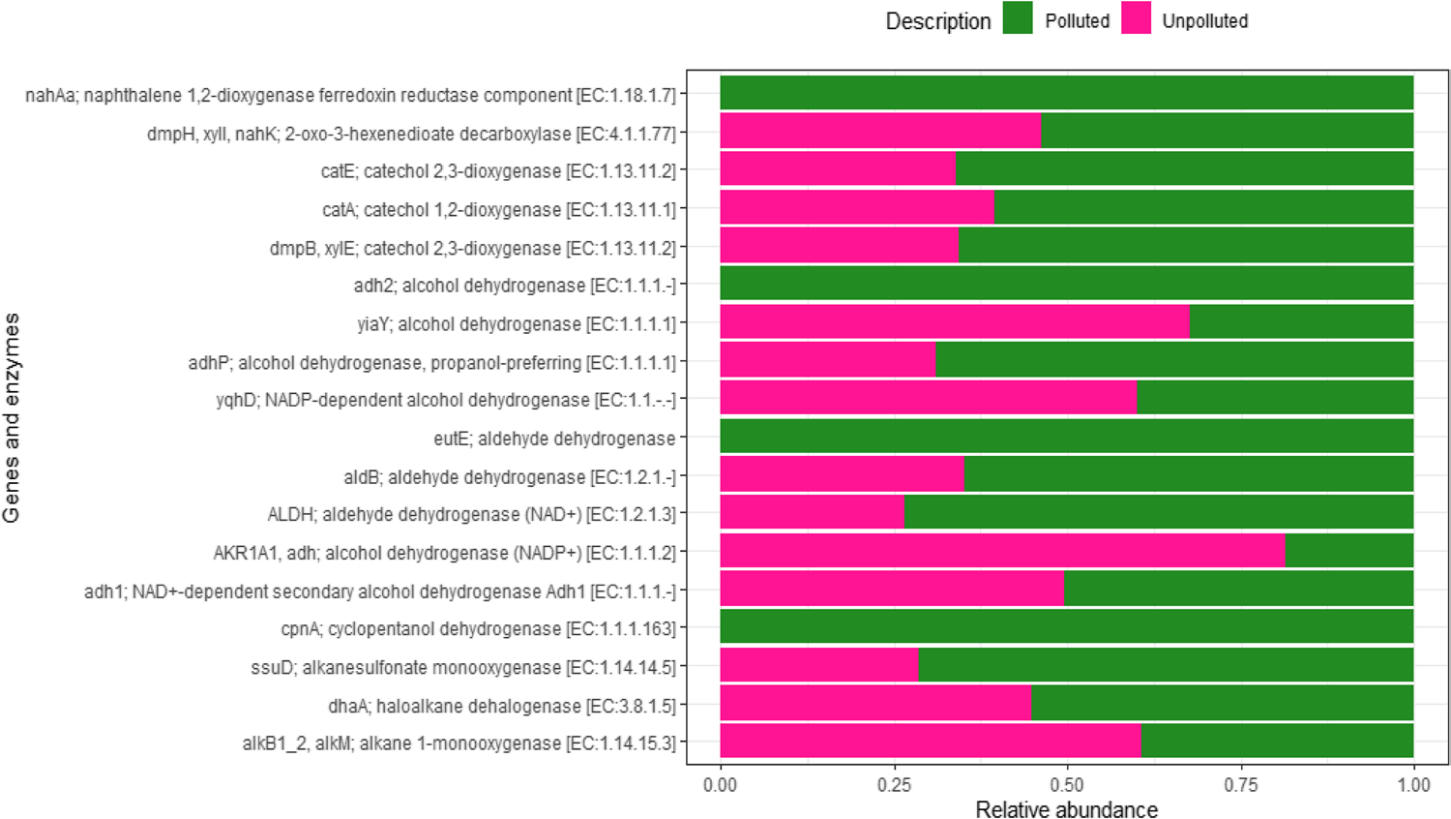
Hydrocarbon catabolic enzymes, genes, and pathways predicted in the polluted and unpolluted soil samples (differences in the overall abundance were significant; *p* = 0.02)

To assess the degradative capabilities and functional traits of bacterial communities in oil-polluted sites, we conducted a comparative analysis of predicted functional genes and enzymes between the polluted and unpolluted sites. Notably, genes like *nahAa* (naphthalene 1,2 dioxygenase ferredoxin reductase), *adh2* (alcohol dehydrogenase), *eutE* (aldehyde dehydrogenase), and *cpnA* (cyclopentanol dehydrogenase) were solely detected in bacterial communities from the oil-polluted samples and were absent in the unpolluted samples. These genes play pivotal roles in catabolic pathways responsible for breaking down aromatic and hydrocarbon compounds. Additionally, various genes involved in multiple metabolic pathways, including *catE*, *catA*, *dmpB*, *aldB*, *ssuD*, *dhaA*, and *alkB*, were identified in both soil communities. However, it is noteworthy that the oil-polluted soils exhibited a relatively higher abundance of these genes compared to the unpolluted soils (Fig. 9).

## Discussion

Soil environments in regions that have been chronically exposed to crude oil contamination are home to a diverse range of microorganisms with unique capabilities for degrading recalcitrant compounds. These microorganisms hold significant potential for application in the oil industry and areas affected by accidental oil spills, offering an effective solution of addressing and reducing the environmental toxicity caused by such incidents through bioremediation techniques. In this study, surface and subsurface soil samples were collected from Gio community, Niger Delta, Nigeria (Fig. 1A), specifically targeting sampling locations significantly affected by oil contamination due to accidental oil spill incidents. The chemical analysis of the soil indicated that the unpolluted soil samples exhibited a relatively higher pH compared to the polluted soil. Notably, the polluted surface soil samples were more acidic than the subsurface soil samples. These findings align with the results of a study by Shaoping et al. (2021), which showed that exposure to petroleum and crude oil compounds tends to decrease soil pH. However, it is worth noting that the extent of pH change may vary depending on the initial soil chemical properties and the concentration of oil present in the soil. When the soil pH becomes acidic, certain reactions, such as the adsorption of heavy metals onto soil organic matter, can become more evident (Jabbarov et al. 2019). This study also revealed that the organic matter content in both surface- and subsurface-polluted soils was relatively high compared to unpolluted soil (Table 1). This increased organic matter content contributed to the adsorption of heavy metals like lead and cadmium from the crude oil, resulting in the enrichment of toxic elements in the soil. This aligns with the observations of Aigberua et al. (2017), where concentrations of heavy metals were noted to be comparatively higher in soils contaminated with crude oil. Notably, the concentration of nickel was higher in unpolluted soil compared to polluted soil (Table 1). However, elevated levels of Ni in different soil layers may be influenced by both natural soil-forming processes and human activities (Pivková et al. 2022).

As expected, both the surface-polluted soil (SPS) and subsurface soil (SPSS) showed significantly higher total organic carbon (TOC) levels compared to the unpolluted soil (UPS). This difference can be attributed to the prolonged exposure of SPS to hydrocarbons in contrast to the relatively limited exposure of UPS. Consistent with the TOC findings, the concentrations of total petroleum hydrocarbons (TPH) were notably higher (*p* < 0.05) in the polluted soils when compared to the unpolluted ones (Fig. 2A). This increase in TOC content within the oil-contaminated areas can be linked to the higher levels of TPH present in the soil. It is important to report that there was a positive correlation between TOC content and TPH content in the soils, highlighting the feasibility of using TOC as an effective tool for monitoring petroleum hydrocarbon contamination due to the fact that TOC encompasses all weight fractions of TPH (Schreier et al. 1999; Kuppusamy et al. 2020). The analysis of carbon chain lengths in polluted soils showed that longer-chain alkanes, particularly C20, were more abundant on the surface soil. Similarly, a study conducted in China petrol station soil samples reported relatively higher concentrations of long-chain alkanes compared to other n-alkane concentrations (Li et al. 2021). This could be because these larger molecules are less mobile than shorter alkanes. In the layers beneath the surface, C18 alkanes were more common, indicating that smaller alkanes might sink deeper into the soil, potentially due to them being more soluble (Chen et al. 2022). The absence of certain long-chain alkanes (C19, C22, C25, C27, C29, C31, C32, and C33) in both the surface and subsurface soils (Fig. 2C) may point to the selective utilization of microbes or the unique characteristics of the oil spill. This study, therefore, focused on the indigenous microbes that catabolize these longer chains. The observed distribution of alkanes in the soil highlights the complex nature of environmental oil contamination, highlighting the influence of physical, chemical, and biological dynamics on the fate of pollutants within the soil matrix (Si-Zhong et al. 2009; Kuppusamy et al. 2020).

In recent era, studies utilizing culture-independent methods have been pivotal in shedding light on microbial populations in extreme environments (Ramganesh et al. 2018; Selvarajan et al. 2018b, a; Sibanda et al. 2018; Ubani et al. 2022). Nevertheless, the role of culture-dependent techniques in extracting and isolating microbes from such extreme conditions remains crucial. These indigenous microbes, having adapted to survive in extreme conditions, could harbor metabolic potential valuable for biotechnological advancement (Ramganesh et al. 2014; Selvarajan et al. 2017b; Sibanda et al. 2017). In this study, an integrated approach of both culture-independent and culture-dependent methods was adopted to explore the capabilities of bacterial communities residing in soils that have been chronically exposed to oil contamination. To isolate bacteria adept at this condition and catabolize long-chain alkanes, soil samples were enriched with crude oil (COE) and paraffin wax (PWE), which are rich in these complex hydrocarbons. Tables 2 and 3 outline the biochemical characteristics of bacterial isolates obtained from enrichment media containing crude oil and paraffin wax. In degradation assays using the DCPIP indicator, approximately 20 bacterial strains were tested for their ability to break down long-chain alkanes. *Marinomonas* emerged as the most proficient in degrading both paraffin wax and crude oil. Previous studies have shown that *Marinomonas* can degrade hydrocarbons to some extent (Melcher et al. 2002; Gontikaki et al. 2018; Gidudu and Chirwa 2022), and a recent study by Zannotti et al. (2023) found this bacterium associated with the Antarctic marine ciliate *Euplotes focardii*, has potential for degrading hydrocarbons commonly found in diesel oil. Moreover, *Marinomonas* can thrive in both marine and terrestrial environments and possesses a genome with sequences encoding various enzymes for benzene and naphthalene degradation (John et al. 2020). Following *Marinomonas*, the *Pseudomonas* demonstrated a strong ability for degrading Hexadecane. This observation aligns with findings from other studies (Zhong et al. 2016; He et al. 2019) that have similarly reported *Pseudomonas* as an efficient degrader of this hydrocarbon. However, it is worth noting that in those previous studies, the degradation rate of n-hexadecane reached 78% after 7 days, whereas our strains exhibited a slightly lower rate of 65% degradation. To provide further insights, we identified the potential genes responsible for long-chain alkane degradation, namely *almA*, in both *Marinomonas* and *Pseudomonas* (Fig. 5A). These genes have the ability to regulate the expression of the *almA* oxygenase, which is known for its involvement in the oxidation of super long-chain alkanes (> C30) (Wentzel et al. 2007). Additionally, the presence of gene *ladA* was confirmed in *Alteromonas*, which displayed a high degradation capacity, closely with *Marinomonas* and *Pseudomonas* (Fig. 5B). Several hydrocarbon-degrading bacteria belonging to the genus *Pseudomonas* or other genera like *Rhodococcus*, *Gordina*, and *Acinetobacter* carries *alkB* gene (Kubota et al. 2008). However, to best of our knowledge, this is the first documented study of the *almA* gene being reported in cultured *Alteromonas* bacteria, as it had previously only been observed in uncultured bacteria from the North Atlantic Ocean (Vázquez Rosas Landa et al. 2023).

Novel technologies continue to expand our understanding of microbial diversity and community structure; this study integrated 16 s amplicon analysis using next-generation sequencing technology along with culture-dependent analysis to comprehensively understand the microbial richness and composition of long-term oil contaminated soil microbial communities. Alpha diversity analysis revealed that surface samples exhibited greater species richness and diversity compared to subsurface samples, similar pattern was observed in bacterial communities of soil contaminated with five different oil refineries in China (Jiao et al. 2016). Bray–Curtis dissimilarity indices indicated distinct differences in bacterial community structures between surface and subsurface soils, with surface samples exhibiting specific variations that led to their separate clustering (Fig. 6). These variations could be traced back to dominant taxa, which accounted for a significant share of the dissimilarity in the overall community, as noted by Shade et al. (2014). Furthermore, despite its significant beta diversity, the minor sub-community had a minimal impact on the differences observed within the overall community structure. This observation aligns with findings by Jiao et al. (2017), who reported similar diversity patterns in soils contaminated by oil. In the taxonomic analysis, *Proteobacteria* emerged as the dominant phylum in both soil sample types (Fig. 7). These findings align with similar results reported by Khan et al. (2018), where *Proteobacteria* were identified as the most abundant phyla among the 45 phyla observed in petroleum-contaminated soils collected from South Australia. This phylum was well-represented by the β-, α-, and γ-*Proteobacterial* classes, a pattern commonly observed in hydrocarbon-amended soils. The prevalence of *Proteobacteria* in these environments is unsurprising, as it is recognized for its ability to metabolize both aliphatic and aromatic compounds. This increased abundance of *Proteobacteria* in response to hydrocarbon pollution has been well documented in various geographical locations (Baruah et al. 2017; Chikere et al. 2019; Cabral et al. 2022; Gao et al. 2022). *Acidobacteria* was the second most dominant phylum followed by *Actinobacteria* in surface soil, while *Firmicutes* and *Actinobacteria* dominated in subsurface soil. These results corroborate a previous study by Sutton et al. (2013), which identified *Proteobacteria, Firmicutes*, *Actinobacteria*, *Acidobacteria*, and *Chloroflexi* as major bacterial groups in long-term diesel-contaminated soil. While *Firmicutes* are well-known for their significant role in breaking down plant polymers, they are not commonly associated with broad metabolic activity involving aromatic and/or aliphatic hydrocarbons (Brzeszcz and Kaszycki 2018).

Analysis of bacterial profiles at genus level revealed that distinct variations among bacterial populations between surface and subsurface oil polluted soil samples. Surface samples exhibited *Burkholderia*, *Pedomicrobium*, *Methyloversatilis*, *Rothia*, *Granulicatella*, *Deflivibacter*, *Rhodobacter*, *Rhodococcus*, *Agrobacterium*, *Novosphingbium*, *Bacillus*, and *Mycobacterium*. In contrast, subsurface samples were characterized with *Sphingomonas*, *Acinetobacter*, *Arcobacter*, *Hyphomicrobium*, *Halomonas*, *Mycoplana*, *Ralstonia*, *Pseudomonas*, and *Lactococcus* (Fig. 8). The genus *Burkholderia* has been recognized for its ability to produce a stable biosurfactant with robust emulsification properties for crude oil, showcasing its potential for use in commercial bioremediation applications for oil-contaminated soils (Almatawah 2017). The obligate methylotrophs within the *Methyloversatilis* group have been noted for their role in the biodegradation of Benazolin-Ethyl in activated sludge and methane production (Cai et al. 2011; Fenibo et al. 2023b). *Pseudomonas* species, as well documented in various studies, can degrade hydrocarbons such as naphthalene, phenanthrene, anthracene, and diesel (Rentz et al. 2004; Shukor et al. 2009; Chikere et al. 2019). These species are also known for producing biosurfactants that enhance the desorption and breakdown of petroleum-derived hydrocarbons (Taccari et al. 2012). *Rhodococcus*, another genus draw attention for its capabilities, efficiently breaks down long-chain n-alkanes and crude oil, making it an advantageous representative for cleaning up areas with significant oil pollution (Whyte et al. 2002). Other genera such as *Sphingomonas*, *Acinetobacter*, *Arcobacter*, *Hyphomicrobium*, and *Halomonas* have also been identified as degraders of hydrocarbons, especially long-chain alkanes (Miyashita 2015; Kumar et al. 2020; Ehiosun et al. 2022). Comparative analysis of potential functional genes and enzymes from both polluted and unpolluted sites highlighted an array of metabolic and catabolic genes implicated in hydrocarbon degradation (Fig. 9). Certain functions (data not shown), such as amino acid transport and metabolism, energy production and conversion, and replication, recombination, and repair, could play a role in adapting to the stress from petroleum hydrocarbons. Previous studies have suggested that having sufficient energy is crucial not only for supporting normal microbial growth and reproduction processes but also for the transportation and metabolism of petroleum hydrocarbons (Xu et al. 2017). In addition, genes such as *nahAa*, *adh2*, *eutE*, and *cpnA* were solely found in the oil-contaminated soil samples. The presence of these genes aligns with recent findings of microbial communities in Arctic Sea Ice, which possess genes capable of producing enzymes that break down a variety of hydrocarbons and aromatic compounds (Peeb et al. 2022). Furthermore, genes that contribute to various metabolic processes like *catE*, *catA*, *dmpB*, *aldB*, *ssuD*, *dhaA*, and *alkB* were present in greater abundance in the contaminated soils. These particular genes are known for their role in the production of enzymes that convert alkanes into alcohols. These alcohols are then further processed into fatty acids and finally broken down via the bacterial β-oxidation pathway (Rojo 2009; Fenibo et al. 2023a). In this study, all genes related to degradation were identified, suggesting that microorganisms capable of degrading petroleum hydrocarbons might display a biogeographic distribution of functional genes associated with degradation. Indeed, these genes linked to the degradation of petroleum hydrocarbons have been identified and detected across different environmental media (Wang et al. 2010; Long et al. 2017). Therefore, considerable efforts should be made to isolate indigenous microbes for a comprehensive exploration of these degradation-related genes. Nevertheless, a comparison of the outcomes from culture-independent analyses reveals that current laboratory isolation efforts have successfully identified only a limited number of predominant members, including *Bacillus, Halomonas, Pseudomonas*, *Rhodococcus*, and *Lactococcus*. These bacteria can thrive under standard laboratory conditions without specialized media or environments. Nonetheless, there is a continuous need to cultivate other significant members in the lab, those that have shown promise in degrading long-chain hydrocarbons.

## Conclusion

In conclusion, this investigation into the microbiota of long-term oil-contaminated soils highlights a diverse array of microorganisms capable of degrading various hydrocarbons. The combined approach, utilizing both culture-dependent and culture-independent methods, has provided a comprehensive understanding of the microbial community structure and potential metabolic functions in these environments. The results indicate a distinct microbial population variation between surface and subsurface soils, with surface soils exhibiting higher microbial richness and diversity. Notably, total organic carbon (TOC) levels in surface-polluted soil (SPS) and subsurface soil (SPSS) range from 5.64 to 5.06%, compared to 1.97% in unpolluted soil (UPS). Similarly, total petroleum hydrocarbons (TPH) levels in SPS and SPSS range from 36,775 ppm to 14,087 ppm, contrasting with UPS at 47,936 ppm. Carbon chain analysis reveals a prevalence of longer-chain alkanes (C20-28) in surface soil. Culture-dependent methods yield 47 bacterial isolates, with 12 strains displaying significant degradation abilities. Culture-independent analysis demonstrates higher species richness in surface samples, dominated by *Proteobacteria*. Specific bacterial genera (e.g., *Pseudomonas*, *Marinomonas*, *Alteromonas*) exhibit 50 to 80% degradation efficiency. Furthermore, the study has also shed light on specific bacterial genera and catabolic genes that contribute to hydrocarbon and long-chain alkanes degradation pathways, offering new insights into the metabolic capabilities present in contaminated sites. However, the results also stress the need for ongoing efforts to isolate and study a broader spectrum of microorganisms under laboratory conditions, to further unlock the bioremediation potential of indigenous soil bacteria that may aid in the development of more effective bioremediation applications for restoring oil-contaminated environments.

### Supplementary Information

Below is the link to the electronic supplementary material.

Supplementary file1 (DOCX 38371 KB)

## Data Availability

The raw sequence data available publicly in the NCBI-SRA under the BioProject ID PRJNA1037324.
