# Supplementary material for: Characterization and identification of long-chain hydrocarbon-degrading bacterial communities in long-term chronically polluted soil in Ogoniland: an integrated approach using culture-dependent and independent methods
Source: Environ Sci Pollut Res Int. 2024 Apr 15;31(21):30867–85. doi: 10.1007/s11356-024-33326-6 (PMC11096258; doi:10.1007/s11356-024-33326-6)
Supplement: Supplementary file 1 — Supplementary file1 (DOCX 38371 KB) [file 11356_2024_33326_MOESM1_ESM.docx]

| **Bacterial species** | **Paraffin oil (POL)** | **Hexadecane (HDX)** | **Heavy crude (HCO)** | **almA gene** | **ladA gene** |
| --- | --- | --- | --- | --- | --- |
| *Lysinibacillus fusiformis (P1)*  *Bacillus pumilus (P16)*  *Pseudomonas aeruginosa (P17) Bacillus cereus (P5)*  *Alteromonas confluentis (P27),*  *Paraglaciecola hydrolytica (C8),*  *Morganella morganii (P31),*  *Stenotrophomonas pavanii (P6),*  *Bacillus pumilus(C1),*  *Bacillus Pumilis (P11),*  *Enterococcus faecalis (C11),*  *Marinomonas atlantica (P35),*  *Providencia rettgerii (P10),*  *Bacillus pumilis (C2),*  *Pseudomonas aeruginosa (P19),*  *Providencia rettgerii (P15),*  *Bacillus altitudinis (C4)*  *Bacillus stratosphericus (C3),*  *Proteus vulgaris (P3)*  *Bacillus pacificus (P13)* | * * *  * * *  * * *  * * *  * * *  * * *  * * *  * * *  * * *  * * *  * * *  * * *  * * *  * * *  * * *  * * *  * * *  * * *  -  - | * * *  * * *  * * *  * * *  * * *  * * *  * * *  * * *  * * *  * * *  * * *  * * *  * * *  * * *  * *  * * *  * * *  -  -  - | ** *  * * *  * * *  * * *  * * *  * * *  * *  * *  * * *  * *  * *  * * *  -  -  -  -  -  -  -  - | +  + | + |

**Characterization and Identification of Long-chain Hydrocarbon-Degrading Bacterial Communities in Long-Term Chronically Polluted Soil in Ogoniland: An Integrated Approach Using Culture-Dependent and Independent Methods**

Amara Ukamaka Okoye^1†^, Ramganesh Selvarajan ^2,3*^, Chioma Blaise Chikere ^1,2^, Gideon Chijioke Okpokwasili^1^, Kevin Mearns^2^

1. Department of Microbiology, Faculty of Science, University of Port Harcourt, Port Harcourt 500272, Nigeria
2. Department of Environmental Science, Florida Campus, University of South Africa, Roodepoort 1709, South Africa
3. Laboratory of Extraterrestrial Ocean Systems (LEOS), Institute of Deep-Sea Science and Engineering, Chinese Academy of Sciences, Sanya 572000, China

**Supplementary Table 1**: Bacterial strains exhibited significant degradation abilities across all enriched media and the presence of potential function genes for long chain degradation.

* * * Significant degradation, * * Moderate degradation, - very weak/no degradation, + -presence of gene

**Supplementary Figure 1:** (A-B) Long term heavily oil polluted area in the Gio community of Niger Delta region, Nigeria, (C-D) the uncontaminated sites within the Gio community illustrating the vegetation-rich farmland and barren upland, respectively.
